# Assessing antifungal stewardship competency among pharmacy students: key findings and implications for clinical practice

**DOI:** 10.3389/fpubh.2026.1717645

**Published:** 2026-06-12

**Authors:** Tahmina Maqbool, Humera Ishaq, Sadia Shakeel, Hina Rehman, Sana Sarfaraz, Halima Sadia, Saira Azhar, Suresh Shanmugham, Ghazala Ishrat, Steward Mudenda, Márió Gajdács, Shazia Qasim Jamshed, Wajid Syed, Majed M. Aljabri

**Affiliations:** 1 Faculty of Pharmacy, Hamdard University, Karachi, Pakistan; 2 Department of Pharmacy Practice, Faculty of Pharmaceutical Sciences, Dow College of Pharmacy, Dow University of Health Sciences, Karachi, Pakistan; 3 Department of Pharmacy Practice, Institute of Pharmaceutical Sciences, Jinnah Sindh Medical University, Karachi, Pakistan; 4 Department of Pharmacology, University of Karachi, Karachi, Pakistan; 5 Department of Pharmacy Practice, Faculty of Pharmacy, Jinnah University for Women, Karachi, Pakistan; 6 Faculty of Pharmacy, Iqra University, Karachi, Pakistan; 7 Department of Pharmacy Practice, School of Pharmacy, IMU (International Medical University), Kuala Lumpur, Malaysia; 8 Department of Pharmaceutics, Faculty of Pharmacy, Salim Habib University (Formerly Barrett Hodgson University), Karachi, Pakistan; 9 Department of Pharmacy, School of Health Sciences, University of Zambia, Lusaka, Zambia; 10 Department of Public Health, Albert Szent-Györgyi Medical School, Faculty of Medicine, University of Szeged, Szeged, Hungary; 11 MTA-SZTE Lendület “Momentum” Anthropogenic Stress and Plant Resilience Research Group, Szeged, Hungary; 12 Department of Clinical Pharmacy, College of Pharmacy, King Saud University, Riyadh, Saudi Arabia; 13 Community and Psychiatric Mental Health Nursing Department, College of Nursing, King Saud University, Riyadh, Saudi Arabia

**Keywords:** antifungal resistance, antifungal stewardship programs, infection, Pakistan, pharmacy students

## Abstract

**Background:**

Antifungal resistance (AFR) is a significant public health concern. It restricts the available treatments for fungal infections, making them more difficult to possibility of treatment and increasing the risk of serious consequences. Pharmacists could significantly promote the rational use of antifungals, lessen their overuse, and support public health initiatives to stop the spread of resistant fungal infections by participating in antifungal stewardship (AFS) programs. Hence, the current study aimed to evaluate undergraduate pharmacy students’ knowledge, attitudes and practices toward AFR.

**Methodology:**

A cross-sectional study was conducted on undergraduate pharmacy students studying their fourth and final year in various public and private universities of Karachi. The study questionnaire consisted of demographic details of students, 10 questions about knowledge, 10 questions about attitude and 10 questions regarding practice. The significance of each variable was evaluated, and the model fit was assessed with −2 Log Likelihood and Nagelkerke *R* Square. Data was analyzed using IBM SPSS version 20.

**Results:**

Out of 1763 students from the 4^th^ year and final year in different universities, 1,325 students responded to the questionnaire, which makes the response rate 75.1%. The students, 75.6% (*n* = 1,002), revealed good knowledge, which improved as the years of education progressed and was statistically significant (*χ*^2^ = 21.06, *p* < 0.001). A positive attitude was observed in 76.1% (*n* = 1,008) students toward AFR and AFS, with statistically significant improvement (*χ*^2^ = 6.94, *p* < 0.001) in incremental study years, being highest in the 5th year (78.4%, *n* = 661/843). Practice score was good in 49% (*n* = 652) students, which was better in the final year (50.1%, *n* = 422/843) compared to the 4^th^ year (47.7%, *n* = 230/482), which was statistically non-significant.

**Conclusion:**

The presented research indicated that most students had an understanding of AFR and AFS, which is encouraging because AFR and AFSs are under- and poorly-researched fields in Pakistan. However, there is still a need to improve the lacking areas where students have low scores regarding AFR and AFS by providing educational interventions. Furthermore, strategies should be implemented to reduce the misuse and overuse of antifungals, especially without a prescription, to reduce AFR in Pakistan.

## 1 Introduction

A public health issue that impacts people, animals, and the environment is antimicrobial resistance (AMR) (1). The impact of AMR includes higher morbidity and mortality rates and difficulties or impossibilities in treating infections (2). By 2050, 10 million people will die each year from antimicrobial resistance (AMR) if this issue is not resolved. It also harms global economic conditions and healthcare expenses (3).

Antifungal resistance (AFR) is considered a growing issue within AMR, yet its incidence has previously been underestimated (4) and needs to be focused on to avoid further increases in mortality rates from AMR. Over 13 million infections and 1.5 million fatalities worldwide occur each year due to fungal pathogens, mostly affecting individuals with weakened immune systems (5).

Most attention, research, and public health strategies have been directed toward resistance to antibacterials and antivirals rather than antifungals, despite increased mortality and costs associated with AFR (6). Days of therapy (DOT) and daily dose (DD) are the parameters used to construct the total antifungal prescriptions (TAP), which have been used to measure antifungal consumption (7). The necessity of AFS programs to avoid and combat unnecessary systemic infections was brought to light by the recent COVID-19 pandemic and the possibility of immunocompromised patients developing systemic fungal infections (8) Due to the increased incidence of fungal infections, particularly in immunocompromised people, the usage of antifungal medications has increased recently (9).

Prescribing antifungals without adhering to National or local guidelines, improper use of drugs, and noncompliance with prescribed therapy by the patients are also contributing factors to AFR. The increased use of fungicides in agriculture is another component that needs to be kept in mind (10). Less availability of newer antifungals in the market, a rise in the trend of immunocompromised people, an increased scattering of fungi and their ability to survive in the environment, unrestricted international travel, and wide variety of possible hosts are other reasons for the increasing trend of AFR (11).

Antifungal resistance raises treatment costs, particularly in low- and middle-income countries (LMICs), and restricts the availability of effective antifungal medicines (12).

According to an assessment of the literature, there are gaps in the fundamental elements of hospital AMS programs in several Asian countries. Considering the increased trend of antifungal use in underdeveloped Asian countries, the AFR would be an additional factor leading to increased mortality.

Several measures must be developed and successfully implemented in countries, especially LMICs, to address the ever-increasing issue of AFR. This should begin with evaluating the key stakeholder groups present understanding, knowledge and attitudes regarding antifungals and AFR to create relevant interventions to lessen issues that have been identified. Secondly, targeted AFS programs should be focused on and educational initiatives should be created and promoted in order to lower AFR. To improve AMS programming in Asian countries, there is an urgent need to find hospital- and country-specific solutions to financing and resource shortages (13).

As evaluated from the research studies, the bulk of research evaluating AMR and antimicrobial usage in Pakistan among patients and ASPs is more focused on antibiotics, antivirals, and anti-TB medications rather than antifungal. Given that a sizable portion of Pakistan’s population suffers from different fungal infections, which might be cutaneous and systemic as well as from immune system-compromising illnesses, which increases the danger of opportunistic fungal infections, this needs to be addressed immediately.

Thus, to close this evidence gap, we first evaluated the knowledge, attitudes, and practices (KAP) of pharmacy students from different universities in Karachi regarding AFR and AFS. This expands upon earlier research on antibiotics and AMR in university students in Karachi (14). This is the first study reported from different universities of Pakistan where the students were evaluated based on their AFR and AFS knowledge. To enhance antifungal utilization going forward, the results can then be utilized to improve university educational programs. Undergraduate pharmacy students were targeted as they are future hospital and community pharmacists, and they should be fully aware of the use and dispensing of antifungals and their possible role in preventing AFR, as we are aware that in Pakistan anti-fungals can be dispensed without a proper prescription from certain chemists shops but community pharmacies associated with tertiary care hospitals where pharmacists are present follow the proper protocol for antifungal dispensing. Hence, we focused on the undergraduate students who are the future pharmacists of Pakistan and are expected to play a role in advising patients regarding AFR.

## 2 Methods

### 2.1 Study design, population and site

As per the STROBE guidelines (15), a cross-sectional study was conducted on undergraduate pharmacy students studying in different public and private sector universities in Karachi. Data was collected from students of the 4th and final professional during May and June 2024. It was considered that the students participating in the research must understand the essence of the questions and give informed consent for further processing of the questionnaire.

### 2.2 Ethical consideration

Ethical approval was taken from the ethical committee at the Faculty of Pharmacy, Hamdard University, Karachi, with the ethical approval number ERC-FOP-2024-004 dated 15th April 2024. A statement of informed consent was added to the Google form. Students had to click the option of “I accept” to continue with the questionnaire.

### 2.3 Eligibility criteria

Students were eligible to participate when they were enrolled in the participating university. They were ready to participate. Students of 1st professional to the third professional were not eligible to participate in the study.

### 2.4 Sample size calculation

The sample size was calculated using Taro Yamane’s formula with the known target (finite) population of 1763 students. This number is the total number of students studying the fourth and final year of pharmacy in different participating universities. Using the formula with a 95% confidence interval and a 5% margin of error, the sample size was calculated to be 326.

### 2.5 Data collection

The data collection tool was adopted from an already published study, with prior approval of the principal investigator (16). The questionnaire was reviewed by the researchers who participated in the study to check the content validity, completeness and understanding of the students from the different universities in Karachi, Pakistan. A pilot study was run to check the clarity of questions in our population, simplicity for both public and private universities relevance, accuracy and robustness in our country and students’ understanding. The participants of the pilot study (*N* = 30) were excluded from the core of the study. There were 6 participating universities, 3 from the public sector and 3 private sector. Data was collected from each participating university by form with all enrolled class students in their class WhatsApp groups. It was ensured that each participating university contributed at least 50% participation. The university that failed to respond to at least 50% participation was excluded (*n* = 1). The resultant Excel data file was then analyzed after conversion into an SPSS file.

### 2.6 Study tool/questionnaire

The study questionnaire was adopted as it is from Mudenda et al. (16) after permission. It was divided into four parts. The first part was demographic details of participants, 2nd part contained 10 questions of knowledge, 3rd part comprised 10 questions about attitude, and the fourth section contained 10 questions regarding practice.

### 2.7 Study measures

Knowledge, attitude and practice were the main outcomes, which were coded as 1 for good and 0 for bad/poor. There were three options for every KAP question that was yes = 2, no = 0 and neutral = 1. Item scores were summed, and consolidated scores for knowledge, attitude and practice were achieved. Continuous data retrieved was then converted into “binary variables” of Good or poor knowledge, positive/negative attitude and good/poor practice with a cut-off of 80% or above to avoid false positive results. The dichotomization at ≥80% was based on the scoring scheme and conceptual considerations. While this threshold reduces false-positive classification, it has not been empirically validated and should be interpreted as operational. The hypothesis was that good knowledge and attitude would positively influence the practice of students.

### 2.8 Statistical analysis

The result of categorical variables was reported as frequencies and percentages. Pearson’s test of correlation was applied to compare the knowledge, attitude and practice scores of students with professional years (i.e., fourth year with final year) and university sectors (i.e., public sector universities with private sector).

All candidate variables (University, Gender, Class (Year of Study), Residence, and Marital Status) were initially included in the full logistic regression model to assess their associations with knowledge, attitude and practice. Variables were coded as categorical, and a full model was constructed. Non-significant variables (*p* > 0.05) were sequentially removed using a backwards stepwise elimination approach. The significance of each variable was evaluated using the Wald test, and the model fit was assessed with −2 Log Likelihood and Nagelkerke *R* Square. Variables with the highest *p*-values were removed one at a time, with the reduced model retaining gender, year of study and university for knowledge, university, gender, and residence for attitude and only Gender for practice as a significant predictor (*p* < 0.001).

The relationship between variables and their manner of association was studied via “generalized structural equation modeling.” A mediation analysis was run to analyze the direct, indirect and total effect on the relationship between knowledge and practice of AFR and AFS, where Attitude was controlled as a mediator. Models were “adjusted” for the year of study/class, and 1,000 rotations of bootstrapping were conducted to improve standard errors. Additionally, it was confirmed that the independent and dependent variables had a linear relationship with or without mediators to fulfill the mediation analysis assumption. Because of the high significance of the result, the multicollinearity model was also applied to observe the correlation between predictors and independent variables. Correlation, tolerance value, variance inflation factor and condition index were tested. All tests were performed using SPSS version 20, and *p* < 0.05 was considered significant.

## 3 Results

### 3.1 Reliability and factor analysis

To assess the internal consistency of the 33-item KAP (Knowledge, Attitude, Practice) questionnaire on AFR and stewardship, Cronbach’s alpha was calculated. The overall Cronbach’s alpha was 0.817, which indicated good internal consistency of the instrument.

Item-total statistics showed that most items had acceptable corrected item-total correlations (>0.30), suggesting good item discrimination. One item (“Over-the-counter antifungal medications cannot lead to AFR”) had a negative corrected item-total correlation (−0.249), which may suggest it was not aligned with the overall scale construct. The Cronbach’s alpha did not substantially improve upon deletion of any item, supporting the retention of all items.

The Kaiser–Meyer–Olkin (KMO) measure of sampling adequacy was 0.866, indicating that the data were well-suited for factor analysis. Bartlett’s test of sphericity was significant (*χ*^2^ = 7231.329, df = 435, *p* < 0.001), confirming that the correlation matrix was not an identity matrix.

Principal Component Analysis (PCA) was performed to explore the underlying structure of the questionnaire. The analysis extracted six components with eigenvalues greater than 1, explaining a cumulative variance of 43.57%. The first component accounted for 16.81% of the variance, followed by 9.93%, 5.45%, 4.17%, 3.83%, and 3.38% for the subsequent components. The factor loadings were moderately distributed across items, supporting the multidimensionality of the instrument.

### 3.2 Demographic characteristics

In the current study, in1325 students responded to the questionnaire circulated to them on social media groups, which makes the response rate 75.1%. The majority of the respondents were female, 70% (*n* = 932). The students, 82% (*n* = 1,088), were residents of the urban locality. Almost all the students 98% (*n* = 1,298) belonged to the age group of 18–25 years. Among participating Student, 42% (*n* = 561) belonged to public sector universities and 58% (*n* = 764) belonged to private sector universities (Table 1).

**Table 1:** Demographic characteristics (*N* = 1,325).

Variable	Category	*N*	(%)
Age	18–25	1,298	98%
	26–33	20	1.5%
	Above 33	7	0.5%
Gender	Male	393	30%
	Female	932	70%
Residence	Rural	153	11.5%
	Rural-urban	84	6.3%
	Urban	1,088	82%
Marital status	Single	1,250	94.3%
	Married	75	5.7%
Year of study	Fourth year	482	36.4%
	Final year	843	63.6%
University type	Public sector	561	42%
	Private sector	764	58%

### 3.3 Knowledge, attitude and practice toward antifungal resistance and stewardship

Table 2 shows that the majority of the students, 75.6% (*n* = 1,002), have good knowledge, which improved as the years of education progressed and was statistically significant (*χ*^2^ = 21.06, *p* < 0.001). Knowledge was found to be higher in 5th-year students (79.7%, *n* = 672/843). A positive attitude toward AFR and AFS was observed in 76.1% (*n* = 1,008) students with statistically significant improvement (*χ*^2^ = 6.94, *p* < 0.001) in incremental study years, being highest in the 5th year (78.4%, *n* = 661/843). Practice score was good in 49% (*n* = 652) students, which was better in the final year (50.1%, *n* = 422/843) compared to the 4th year (47.7%, *n* = 230/482), which was statistically non-significant.

**Table 2:** Student’s knowledge, attitude and practice scores.

Variable	Total (*n* = 1,325)	Year of study/class		Sig	University		Sig
		4th year (*n* = 482)	5th year (*n* = 843)		Public sector (*n* = 561)	Private sector (*n* = 764)	
Knowledge *n* (%)							
Good	1,002 (75.6%)	330	672	*χ*^2^ = 21.06 <0.001	482	521	*χ*^2^ = 55.94 *p* < 0.001
Poor	323 (24.4%)	152	171		79	244	
Attitude *n* (%)							
Positive	1,008 (76.1%)	347	661	*χ*^2^ = 6.94 *p* = 0.008	470	538	*χ*^2^ = 31.72 *p* < 0.001
Negative	317 (23.9%)	135	182		91	226	
Practice *n* (%)							
Good	652 (49%)	230	422	*χ*^2^ = 0.67 NS	266	386	*χ*^2^ = 1.25 NS
Poor	673 (50.8%)	252	421		295	378	

Table 2 also shows the comparison between public and private universities, revealing that students belonging to the public sector, 85.9% (482/561), have good knowledge as compared to private sector students, 68.2% (*n* = 521/764); the difference was found statistically significant. Good attitude was also shown in public sector students, 83.8% (*n* = 470/561), when compared to private sector students, 70.4% (*n* = 538/764), which was found to be statistically significant (*χ*^2^ = 31.77, *p* < 0.001). Good practice was statistically non-significant between both sector universities (Table 2).

It was observed that 94% (*n* = 1,246) of students knew the definition of antifungals and 93.4% (*n* = 1,236) were able to name the drugs of different groups. Around half of the students, 51.7% (n = 685), knew that over the counter (OT) use of antifungal agents may cause AFR. The mass population, 90.9% (*n* = 1,204), knew that AFR is triggered by improper use of antifungal agents and 78.8% (*n* = 1,044) knew that prolonged illness and even death can be encountered due to AFR. Around 90%believed that this problem could be overcome by proper control measures (Table 3).

**Table 3:** Students’ responses.

Domain + questions	Yes *n* (%)	No *n* (%)	Do not know *n* (%)
Knowledge			
“Antifungal resistance is a phenomenon where fungi become less responsive to antifungal medications”	1,246 (94%)	39 (2.9%)	40 (3%)
“Fluconazole, amphotericin B, and itraconazole are examples of common antifungal medications”	1,237 (93.4%)	31 (2.3%)	57 (4.3%)
“Misuse or overuse of antifungal medications contributes to the development of antifungal resistance”	1,204 (90.9%)	59 (4.5%)	62 (4.7%)
“Antifungal resistance could lead to prolonged illnesses and higher mortality rates.”	1,044 (78.8%)	96 (7.2%)	185 (14%)
“Only bacterial infections can develop resistance; fungal infections cannot”	177 (13.4%)	1,012 (76.4)	136 (10.3%)
“It’s important to complete the full course of prescribed antifungal treatment, even if symptoms improve earlier.”	1,166 (88%)	68 (5.1%)	91 (6.9%)
“Antifungal stewardship programs aim to improve the use of these drugs”	1,189 (89.7%)	54 (4.1%)	82 (6.2%)
“Over-the-counter antifungal medications cannot lead to antifungal resistance.”	364 (27.5%)	685 (51.7%)	276 (20.8%)
“Patient adherence to the prescribed antifungal regimen is crucial for effective treatment.”	1,148 (86.6%)	74 (5.6%)	103 (7.8%)
“Regular diagnostics are not necessary when prescribing antifungal treatment.”	447 (33.7%)	664 (50.1%)	214 (16.2%)
Attitude			
“Antifungal resistance is a significant public health concern”	1,114 (84.1%)	84 (6.3%)	127 (9.6%)
“The current training and education about the proper use of antifungals and antimicrobial resistance are sufficient”	614 (46.3%)	558 (42.1%)	153 (11.5)
“It is okay to prescribe antifungal medication even without a confirmed fungal infection”	227 (17.1%)	1,001 (75.5%)	97 (7.3%)
“All healthcare students should participate in AFS programs.”	1,178 (88.9%)	56 (4.2%)	91 (6.9%)
“Patient non-compliance with antifungal medicines contributes to the occurrence of antifungal resistance”	989 (74.6%)	151 (11.4%)	185 (14%)
“Overuse or misuse of antifungal medications in healthcare practices is a public health concern.”	1,144 (86.3%)	76 (5.7%)	105 (7.9%)
“The proper use of antifungal medicines is a critical part of effective patient care.”	1,193 (90%)	70 (5.3%)	62 (4.7%)
“It’s necessary to discuss antifungal resistance and its implications with patients.”	1,219 (92%)	41 (3.1%)	65 (4.9%)
“I believe that more research is needed in the field of antifungal resistance”	1,201 (90.6%)	40 (3%)	84 (6.3%)
“Preventive measures, such as infection control and prophylaxis, are important in managing antifungal resistance.”	1,123 (84.8%)	67 (5.1%)	135 (10.2%)
Practice			
“I bought antifungal medicines without a prescription.”	418 (31.5%)	797 (60.2%)	110 (8.3%)
“When my family/friend is sick, I recommend buying antifungals.”	199 (15%)	984 (74.3%)	142 (10.7%)
“I use antifungals because of advice from friends and family”	219 (20.9%)	992 (66.5%)	114 (8.6%)
“I use antifungals when I have a urinary tract infection.”	277 (20.9%)	881 (66.5%)	167 (12.6%)
“I use antifungal medicines when I have a cold”	158 (11.9%)	1,081 (81.6%)	86 (6.5%)
“I seek additional education or training on antifungal medications and resistance”	950 (71.7%)	204 (15.4%)	171 (12.9%)
“Prescribing physicians and students are the only professionals who need to understand antifungal stewardship.”	492 (37.1%)	694 (52.4%)	139 (10.5%)
“I participate in AFS and awareness programs.”	753 (56.8%)	365 (27.5%)	207 (15.6%)
“I keep myself updated about the latest research and guidelines regarding antifungal medications and antimicrobial resistance”	793 (59.8%)	235 (17.7%)	297 (22.4%)
“Formal teaching on the proper usage of antifungals among healthcare students is an intervention that may minimize the phenomenon of antifungal resistance.”	1,167 (88.1%)	48 (3.6%)	110 (8.3%)

Many pharmacy students (84.1%, *n* = 1,114) were aware that AFR is a health hazard but only 42.1% (*n* = 558) were concerned that the training regarding antifungal agents at our teaching institutions is not enough. Around 75% of the students do not recommend the use of antifungals to their family and friends and 71.7% (*n* = 950) of students reported that they have undergone additional training on antifungal stewardship (Table 3).

### 3.4 Predictors affecting knowledge, attitude and practices of antifungal resistance (AFR) and stewardship (AFS)

For knowledge, attitude and practice variables initially five predictors namely year of study, university, sex, marital status and residence were included to generate a full model. Knowledge-full model showed an 8.1% variance outcome. The full model showed residence and marital status as non-significant predictors. Therefore, to achieve a more parsimonious model, a re-run was conducted without these predictors, and the reduced model explained 7.6%. To achieve a more parsimonious model, a re-run was conducted without these predictors, and the reduced model explained 7.6. Therefore, to achieve a more parsimonious model, a re-run was done without these predictors, and the reduced model explained 7.6% of the variance. The final model showed public university students had 61% higher odds, females had 26% higher odds and final-year students had 40% higher odds of good knowledge compared to their respective counterparts.

For attitude full model showed a 7.4% variance in outcome. Here, class and marital status were found to be non-significant predictors, so after removing the predictors final model showed 7.3% explanatory power which was almost equal to the full model. Here, public sector university students showed 51% higher odds, females showed 43% higher odds and urban residents showed 91% higher odds for a positive attitude. For practice, the full model showed a 1.8% variance in outcome, which was quite low. Here full model only showed gender as a significant influencer on practice. Therefore, all the other non-significant predictors were removed and the parsimonious model showed a 1.3% variance in outcome. Males were found to have 38.4% lower odds for good practice compared to female participants (see Table 4).

**Table 4:** Predictors of good KAP among undergraduate students of pharmacy.

Variable	Knowledge		Attitude		Practice	
	AOR (95% CI)	*p*-value	AOR (95% CI)	*p*-value	AOR (95% CI)	*p*-value
Year of study/class						
4th prof	Ref					
5th prof	1.399 (1.069–1.830)	**0.015**				
Sex						
Female	Ref		Ref		Ref	
Male	0.740 (0.562–0.973)	**0.031**	0.568 (0.433–0.745)	**<0.001**	0.652 (0.513–0.827)	**<0.001**
Residence						
Rural			Ref			
Rural-urban			1.724 (1.189–2.501)	**0.004**		
Urban			1.940 (1.205–3.122)	**0.006**		
University (sector)						
Public	Ref					
Private	0.387 (0.289–0.519)	**<0.001**	0.479 (0.363–0.632)	**<0.001**		

AOR, adjusted odds ratio; 95% CI, 95% confidence interval.

Bold indicates statistical significance < 0.05; Good knowledge, positive attitude and good practice were scores of 80% and above.

Marital status data was removed from the table as it did not appear as a predictor for any of the variables.

### 3.5 Mediation analysis for KAP

Knowledge was positively associated with practice, and this relationship was partially mediated by Attitude. In the total-effect model, higher knowledge was associated with greater odds of good practice [OR = 0.791, 95% CI (0.760–0.835), *p* < 0.001]. Knowledge significantly predicted Attitude (path a: unstandardized *B* = −0.516, SE = 0.037, *p* < 0.001), indicating that each unit increase in knowledge score was associated with an average increase of 0.597 points in the attitude score.

When both Knowledge and Attitude were entered into the logistic regression predicting good Practice, Attitude remained a significant independent predictor [path b: adjusted OR = 0.760, 95% CI (0.719–0.803), *p* < 0.001], while the direct effect of Knowledge on Practice was attenuated but remained significant [c′: adjusted OR = 0.924, 95% CI (0.868–0.985), *p* = 0.014]. Bootstrap mediation analysis (*n* = 1,000) showed a statistically significant indirect effect of Knowledge on Practice via Attitude [indirect effect = 0.142; 95% bootstrap CI (0.107–0.177)], consistent with partial mediation.

In summary, these results indicate that higher knowledge is associated with better practice both directly and indirectly through improved attitudes; the mediation effect was statistically significant but partial, suggesting that other unmeasured factors may also contribute to the Knowledge → Practice pathway.

Results remained consistent when adjusted with the study years/class. Due to the presence of very low variance multicollinearity test was conducted. Four constructs of multicollinearity were analyzed, and none of them showed evidence of the presence of any multicollinearity, as all the variables showed a Variation inflation factor (VIF) ranging from 1.024 to 1.215 (Tables 5, 6).

**Table 5:** Mediation analysis for the influence of knowledge toward practice regarding AFS and AFR.

Effects	Mediator (attitude)	
	AOR (95% CI)	*p*-value
Total	0.791 (0.750–0.835)	<0.001
Indirect	1.39 (1.113–1.194)	<0.001
Direct	0.924 (0.868–0.985)	0.014

**Table 6:** Multicollinearity statistics with the dependent variable being practice.

Coefficients			
Model		Collinearity statistics	
		Tolerance	VIF
1^a^	Attitude	0.823	1.215
	Knowledge	0.836	1.196
	University	0.896	1.117
	Class/year of study	0.911	1.097
	Residency	0.977	1.024

^a^Dependent variable: practice.

## 4 Discussion

The present study revealed the prospective pharmacists’ understanding of the phenomenon of AFR and AFS. Even though AFR continues to develop, there is not an abundance of information about the pharmacy students’ comprehension of the subject matter. To our knowledge, this is the first study conducted in Pakistan to evaluate undergraduate pharmacy students’ insight into AFR. Of the students that took part, the majority of pharmacy students were observed to be well-comprehended about AFR and AFS, which is consistent with earlier research studies that evaluated students’ familiarity with AMR (17–19). The higher level of awareness noticed in our study might be due to the information gained by pharmacy students during their education and clerkship. It is also encouraging to note that the majority of pharmacy students were aware of the terminology AFS, and AFS-promoting variables, and consider AFR as a major public health concern. These results support earlier research in which students were conversant with the implications of AMR, predisposing factors, and stated antimicrobial susceptibility factors as means of addressing this public health issue (20). In the current study, around 90% of the students acknowledged that AFS programs could improve the rational use of these drugs by making sure that antifungal medications are provided correctly, at the proper dose, and for the right duration. A Japanese study reported that the successful implementation of AFSPs enhances patient outcomes, reduces side effects, and maintains the effectiveness of currently available antifungal treatments by encouraging evidence-based practices (21). Another study reported that the integration of an AFS program enhances the appropriateness of antifungal therapy (22).

Fungal pathogens are a major threat to healthcare due to their rapid evolution and this consequently increases AFR; hence, the treatments for fungal diseases are becoming less effective. In addition, failure to address a patient’s condition can result in a prolonged disease and sometimes death. Therefore, researchers are becoming increasingly concerned about changes in fungal evolution and their transmission (23). Around 80% of the students in the present study were familiar with AFR can lead to prolonged illness because resistant fungal infections are more difficult to treat and demand longer or more vigorous therapies. Patients may face worsening illnesses as treatment options become more limited, which could result in longer hospital stays and more complex medical care. It was observed in the present study that students of private sector universities were less likely to have sound knowledge as compared to public sector university students. Similarly, students of public sector universities and those in their final year, as opposed to those in private sector universities and 4th year students were likely to report having an optimistic attitude. Another study reported that fourth-year students were less likely to indicate favorable attitudes than second-year students. Additionally, fourth-year students were less likely to indicate good practices than second-year students regarding AFR and AFS (16).

The current investigation indicated more optimistic attitudes than another study conducted on Chinese students toward AMR (24). Even though the majority of students in our survey had positive opinions, 42.1% of them said that the education they had received on antifungals, AFR, and AFS was insufficient. The findings are comparable to the Colombian study, where the majority of students thought their existing education practices did not provide them with enough information about AMR and AMS (18). In the present study, more than 90% of the students were acquainted that it’s essential to discuss AFR and its implications with patients to make sure that they understand the significance of adhering to prescribed medicines and the possible consequences of non-adherence. Patients who are informed about the risks of resistance are more likely to take their medications responsibly and are less likely to self-medicate or early conclude their treatment, both of which can exacerbate resistance (25).

It was encouraging to note that more than 70% of the students in the present study were seeking further educational opportunities on AFR to gain a deeper understanding of the complications associated with treating fungal infections. Adequately teaching pharmacy students about antifungal pharmacotherapy is important as fungal infections are common and can range from superficial to life-threatening conditions, especially in patients with impaired immune systems. Pharmacists can treat patients more effectively if they are aware of AFR and the side effects of these drugs. Furthermore, the pharmacodynamics and pharmacokinetics of antifungal drugs are crucial for pharmacists to comprehend because they frequently have intricate modes of action (22). Nguyen et al. (26) conducted a clinical audit of fluconazole prescribing and found that pharmacy students contributed positively to the antifungal audit and promoted the good stewardship practices for the hospital.

In the current study, students’ practice scores were good and improved with the progressive year of study; however, the practice score was not significantly associated with the students’ year of study and their universities. Contrary to this, another study reported that the good knowledge of respondents was significantly associated with good practice (16). Notably, it was observed that 31.5% of the students had bought antifungals without a prescription. Another study reported that 38.2% of students claimed to use antifungal medications, and the primary drivers of self-medication with antifungals were the belief that their health issue was minor and their fear of visiting a physician (27). Mushi et al. (28), stated that more than half of the respondents (55.5%) reported using over-the-counter antifungals for self-medication. A Danish study reported that the majority of pharmacy employees encouraged customers to start therapy with over-the-counter topical terbinafine, and very few were aware of the present issue of AFR (29). Antifungals are prescription drugs, so it’s essential to avoid self-diagnosis and self-medication, even though it would seem beneficial to suggest them to ill family members or friends. There are differences in the type and severity of fungal infections, and using antifungals incorrectly might worsen the condition, increase resistance, and even be harmful. In the present study, only 15% of the students recommended buying antifungals for their friends and family, which is parallel to another study (16). Around 70% of students in Saudi Arabian research believed that self-medication was unsafe (30). Despite high levels of knowledge and positive attitudes, less than half of the students demonstrated good practices, highlighting a clear intention–practice gap. This finding is particularly concerning, as it suggests that cognitive awareness alone may not translate into appropriate AFS behaviors without structured experiential training and regulatory reinforcement.

The observed gender differences in practice and the higher knowledge and attitude scores among public-sector university students may reflect variations in curricular rigor, clinical exposure, and access to structured stewardship-related training. Public-sector institutions often have closer affiliations with tertiary care hospitals, potentially providing greater exposure to antimicrobial stewardship concepts. Gender-related differences may also be influenced by learning styles, risk perception, and adherence to professional norms; however, these interpretations should be considered exploratory in the absence of qualitative or curriculum-level data.

The current research reported that more than half of the students participated in AFS and awareness programs and kept themselves well-informed about the most recent findings and recommendations about antifungal drugs. Contrary to this another study reported that a meager percentage of students stated that to were updated about the AFS (16). Pharmacy students must stay updated with AFR because new resistant strains may restrict available treatments, making infections more difficult to treat. Pharmacists can choose the most effective antifungal treatments and modify treatment plans as necessary by having a thorough understanding of current resistance patterns. They can effectively tackle the threat that resistant fungi could impose on public health by staying informed and achieving the best possible outcomes for their patients (22).

In Pakistan, the misuse and overuse of antifungals is an increasing concern, contributing to the rise in AFR. Many patients opt to self-medicate or depend on over-the-counter antifungal drugs without sufficient medical direction. These low-priced drugs combined with their misuse might lead to inadequate dosage, unfinished treatment regimens, and the emergence of resistant fungal strains (31). To address this issue and encourage appropriate use of these drugs the Drug Regulatory Authority of Pakistan (DRAP) should contribute actively in controlling the approval, standard, and accessibility of antifungal drugs. By promoting the rational use of these therapies, DRAP must make sure that only safe and effective antifungal drugs are licensed and available for use, contributing to the fight against resistance. Furthermore, DRAP could prevent the overuse and abuse of antifungals by its regulatory control, which are major contributors to the emergence of fungal resistance. Additionally, the Ministry of Health should promote AFSPs and advocate for the advancement of AFR research and awareness campaigns.

The present findings revealed that the knowledge and attitudes of pharmacy students seem promising; however, it’s essential to construe these outcomes cautiously. Being students, they are not yet authorized pharmacists and are deficient in the real-world professional expertise that could assist them in decision-making. Our study demonstrates a striking intention-practice gap: while knowledge and attitudes regarding [topic] were high among medical students, actual practice was markedly lower (49%). This gap was significantly influenced by gender and type of institution, with female students and those from public sector institutions more likely to adhere to recommended practices. These findings suggest that knowledge alone is insufficient to ensure correct behavior and that contextual factors—such as cultural norms, institutional culture, and curriculum emphasis—play a critical role. The discrepancy highlights the need for targeted interventions that focus on behavioral translation of knowledge, rather than simply reinforcing theoretical awareness.

To address these gaps, targeted, evidence-based interventions are recommended; first improvement and review of pharmacy curriculum in context with the AFR and its practical applications followed by interactive case-based learning with simulations from real world case scenarios. Workshops and mentorship programs to address practice adherence. Follow-up assessments after implementation of recommended upgradation in the curriculum especially tailored in institutional and cultural context.

These specific recommendations can contribute to the development of a more thorough and robust grasp of the AFR in pharmacy education. The present findings indicate generally favorable knowledge and attitudes toward AFR and stewardship; however, these results must be interpreted with caution. The categorization of “good” knowledge and attitudes was based on an arbitrary cut-off that was not psychometrically validated, which may overestimate true competence and obscure variability within groups. Additionally, the practice domain relied on self-reported behaviors that may not reflect evidence-based AFS and are susceptible to social desirability bias. The low proportion of variance explained by regression models further suggests that key determinants—such as quality of teaching, clinical exposure, and institutional stewardship culture—were not captured. Therefore, while the descriptive findings are encouraging, they should not be interpreted as confirmation of clinical proficiency or stewardship readiness without validation using objective or longitudinal measures.

The present study provides a comprehensive assessment of undergraduate pharmacy students’ KAP regarding AFR and AFS in Pakistan. To our knowledge, this is the first study in the country to evaluate pharmacy students’ insight into these areas. Overall, the majority of students demonstrated strong knowledge and positive attitudes toward AFR and AFS, consistent with prior research on antimicrobial resistance awareness among health students (17–20). Most students recognized AFR as a significant public health concern and were familiar with AFS concepts, mechanisms, and the importance of evidence-based antifungal use. Approximately 90% acknowledged that AFS programs could improve rational antifungal therapy by ensuring appropriate drug selection, dosing, and duration, aligning with international evidence demonstrating the effectiveness of stewardship programs in improving patient outcomes and reducing side effects (21, 22).

Despite these encouraging findings, the study revealed a substantial intention-practice gap. Less than half of the students demonstrated appropriate AFS practices, and around 30% reported self-medicating with antifungals without a prescription. This gap suggests that high knowledge and positive attitudes do not automatically translate into correct behavior, highlighting the need for interventions that focus on behavioral application rather than solely theoretical understanding. Gender and institutional differences were notable, with female students and those in public-sector universities exhibiting higher practice adherence. Such differences may reflect variations in curriculum rigor, clinical exposure, or institutional emphasis on stewardship.

Students’ perceptions are still evolving and may continue to change as they progress through their education and clinical training. While final-year students demonstrated better knowledge and attitudes in some domains, they did not always show superior practice, suggesting that experiential learning and structured supervision are crucial for translating knowledge into action. These findings are consistent with prior reports indicating that knowledge alone is insufficient to ensure appropriate stewardship behavior (16, 24–30).

Fungal pathogens pose a significant threat to patient health due to rapid evolution and rising resistance, making antifungal treatment increasingly challenging. In Pakistan, misuse and overuse of antifungals, including self-medication and easy access to over-the-counter drugs, exacerbate this issue (31). Students recognized the potential consequences of resistance, such as prolonged illness, limited treatment options, and increased healthcare complexity, emphasizing the importance of incorporating practical, evidence-based stewardship training into the curriculum.

### 4.1 Limitations

Several limitations should be considered. First, dichotomizing continuous KAP scores may reduce variability, and the ≥80% cut-off, although consistent with KAP conventions, was not psychometrically validated. Second, the study did not capture contextual variables such as socioeconomic status, prior AFS exposure, teaching quality, or resource availability, which may explain the low variance in regression models. Third, the reliance on self-reported practice data introduces potential social desirability bias. Finally, the study was conducted in pharmacy institutes within a single city (Karachi), limiting generalizability to other regions in Pakistan. Despite these limitations, the findings provide valuable insights for curriculum development, targeted educational interventions, and policy initiatives aimed at improving antifungal stewardship.

## 5 Conclusion

The majority of pharmacy students demonstrated good knowledge and positive attitudes toward AFR and AFS, reflecting promising awareness in a context where these topics remain under-researched in Pakistan. However, the study revealed a clear intention-practice gap, with less than half of students translating knowledge into appropriate antifungal practices. Targeted educational interventions—including curriculum enhancement, interactive case-based learning, mentorship, and structured feedback—are needed to equip future pharmacists with the skills necessary for rational antifungal use. Implementing these strategies can improve clinical decision-making, reduce inappropriate antifungal use, and ultimately contribute to better patient care and public health outcomes.

### 5.1 Recommendations

Based on the study findings, the following evidence-linked recommendations are proposed:

1. Curricular review and integration: Pharmacy curricula should be updated to include comprehensive modules on AFR mechanisms, global epidemiology, and evidence-based countermeasures. Emphasis should be placed on linking theoretical knowledge with real-world practice.
2. Interactive case-based learning: Implement simulations and case studies that mimic real-life scenarios, including patient-specific characteristics and resistance patterns, allowing students to practice decision-making and appropriate antifungal use.
3. Mentorship and behavioral programs: Develop peer-led workshops, mentorship opportunities, and clinical exposure programs to address observed gender and institutional differences in practice adherence.
4. Monitoring and feedback: Incorporate follow-up assessments, audits, and immediate feedback to reinforce proper antifungal practices and track improvements over time.
5. Contextual adaptation: Tailor interventions to institutional and cultural contexts, recognizing that approaches effective in private-sector institutions may require modification for public-sector universities.
6. Professional development and awareness: Encourage students to participate in AFS programs and stay updated on evolving resistance trends to ensure readiness for future clinical responsibilities.

These targeted interventions aim to bridge the observed intention–practice gap, equipping prospective pharmacists with the knowledge, skills, and behaviors required for effective antifungal stewardship.

## Data Availability

The raw data supporting the conclusions of this article will be made available by the authors, upon reasonable request.
